# Mammalian TRIM67 Functions in Brain Development and Behavior

**DOI:** 10.1523/ENEURO.0186-18.2018

**Published:** 2018-06-14

**Authors:** Nicholas P. Boyer, Caroline Monkiewicz, Shalini Menon, Sheryl S. Moy, Stephanie L. Gupton

**Affiliations:** 1 Curriculum in Neurobiology, University of North Carolina at Chapel Hill, Chapel Hill, NC 27599; 2 Department of Cell Biology and Physiology, University of North Carolina at Chapel Hill, Chapel Hill, NC 27599; 3 Department of Psychiatry, University of North Carolina at Chapel Hill, Chapel Hill, NC 27599; 4 Carolina Institute for Developmental Disabilities, University of North Carolina at Chapel Hill, Chapel Hill, NC 27599; 5 Neuroscience Center, University of North Carolina at Chapel Hill, Chapel Hill, NC 27599; 6 Lineberger Comprehensive Cancer Center, University of North Carolina at Chapel Hill, Chapel Hill, NC 27599

**Keywords:** commissure, DCC, hippocampus, knock-out, striatum, TRIM67

## Abstract

Class I members of the tripartite motif (TRIM) family of E3 ubiquitin ligases evolutionarily appeared just prior to the advent of neuronal like cells and have been implicated in neuronal development from invertebrates to mammals. The single Class I TRIM in *Drosophila melanogaster* and *Caenorhabditis elegans* and the mammalian Class I TRIM9 regulate axon branching and guidance in response to the guidance cue netrin, whereas mammalian TRIM46 establishes the axon initial segment. In humans, mutations in TRIM1 and TRIM18 are implicated in Opitz Syndrome, characterized by midline defects and often intellectual disability. We find that although TRIM67 is the least studied vertebrate Class I TRIM, it is the most evolutionarily conserved. Here we show that mammalian TRIM67 interacts with both its closest paralog TRIM9 and the netrin receptor DCC and is differentially enriched in specific brain regions during development and adulthood. We describe the anatomical and behavioral consequences of deletion of murine *Trim67*. While viable, mice lacking *Trim67* exhibit abnormal anatomy of specific brain regions, including hypotrophy of the hippocampus, striatum, amygdala, and thalamus, and thinning of forebrain commissures. Additionally, *Trim67^−/−^* mice display impairments in spatial memory, cognitive flexibility, social novelty preference, muscle function, and sensorimotor gating, whereas several other behaviors remain intact. This study demonstrates the necessity for TRIM67 in appropriate brain development and behavior.

## Significance Statement

Class I tripartite motif (TRIM) E3 ubiquitin ligases play important roles in neuronal development and function, some of which occur downstream of netrin. TRIM67 is the most evolutionarily conserved of this class and is developmentally regulated and brain enriched. Deletion of murine *Trim67* results in malformations of a subset of subcortical brain regions and of cortical and subcortical myelinated fiber tracts, as well as deficits in spatial memory, motor function, sociability and sensorimotor gating. Interactions between TRIM67 and both the netrin receptor DCC and TRIM9, and abnormalities in netrin-sensitive brain regions are noted. We conclude that TRIM67 is critical for appropriate brain development and behavior.

## Introduction

E3 ubiquitin ligases mediate covalent attachment of ubiquitin to specific substrates. This posttranslational modification targets substrates for degradation or affects substrate localization, interactions, or function (Komander and Rape, 2012; Ciechanover, 2015). In developing neurons we have found that specific non-degradative ubiquitination events alter cytoskeletal dynamics and signaling pathways during axonal morphogenesis (Menon et al., 2015; Plooster et al., 2017). Although the expression patterns and functions of many ubiquitin ligases are not known, many are brain enriched (Deshaies and Joazeiro, 2009) and implicated in neurodevelopmental and neurodegenerative diseases (Anuppalle et al., 2013; Atkin and Paulson, 2014). Specific members of the tripartite motif (TRIM) family of E3 ligases are brain enriched and regulate neuronal development and function (Tocchini and Ciosk, 2015; Fatima et al., 2016; Olsson et al., 2016; Jin et al., 2017); however, the functions and expression patterns of many TRIM proteins are unknown.

TRIM proteins are subdivided into classes based on their carboxy-terminal domains (Short and Cox, 2006). The mouse and human genomes contain six Class I TRIMs, comprising three pairs of paralogs. Mutations in the TRIM1 and TRIM18 pair are associated with X-linked Opitz syndrome (Quaderi et al., 1997; Cainarca et al., 1999; Geetha et al., 2014), characterized by midline birth defects, and frequently intellectual and motor disabilities. TRIM46 is required for formation of the axon initial segment (van Beuningen et al., 2015), and mutations in its closest paralog TRIM36 are associated with anencephaly (Singh et al., 2017). TRIM9 and TRIM67 were previously described as the most evolutionarily conserved pair of Class I TRIMs (Short and Cox, 2006). TRIM9 localizes to Parkinsonian Lewy bodies (Tanji et al., 2010), may be linked to atypical psychosis (Kanazawa et al., 2013), and regulates netrin-dependent axon guidance and branching through the receptor DCC (Winkle et al., 2014; Menon et al., 2015; Plooster et al., 2017). *Trim9* deletion is also associated with aberrant migration, morphogenesis, and synapse organization of adult-born neurons in the murine dentate gyrus, and severe deficits in spatial learning and memory (Winkle et al., 2016). A single study has demonstrated that TRIM67 is expressed in the mouse and human brain and regulates neuritogenesis in a mouse neuroblastoma cell line (Yaguchi et al., 2012), although its role in neurons and *in vivo* is unknown.

In *Drosophila melanogaster* and *Caenorhabditis elegans*, there is a single Class I *Trim* (Short and Cox, 2006). Loss of this TRIM in either organism phenocopies the axon guidance and branching defects that occur on loss-of-function of invertebrate orthologs of the axon guidance cue netrin or its receptor DCC (Alexander et al., 2010; Hao et al., 2010; Morikawa et al., 2011). Sequence comparison of the *D. melanogaster* to human Class I TRIMs suggested that TRIM9 was the closest mammalian ortholog (Short and Cox, 2006; Morikawa et al., 2011). In mice, loss of *Ntn1* or *Dcc* causes agenesis of the corpus callosum and hippocampal commissure, as well as other axon guidance and branching defects (Serafini et al., 1996; Fazeli et al., 1997; Bin et al., 2015). In contrast to invertebrates, we have shown that deletion of murine *Trim9* does not phenocopy loss of *Ntn1* or *Dcc* and in some cases exhibits an opposite, gain-of-function phenotype, including increased axon branching and thickening of the corpus callosum (Winkle et al., 2014; Menon et al., 2015). This suggested TRIM9 was not the functional ortholog of the invertebrate Class I TRIM.

Here, we show that TRIM67 interacts with its paralog TRIM9 and the netrin receptor DCC. We found that deletion of murine *Trim67* results in impairments in spatial memory, cognitive flexibility, social novelty preference, muscle function, and sensorimotor gating. Histologic analysis demonstrated decreases in the size of a specific subset of brain regions in *Trim67^−/−^* mice, including the hippocampus, caudate putamen (CPu), thalamus, and amygdala. Additionally, there were decreases in the size of the internal capsule and the dorsal commissures, including the corpus callosum and hippocampal commissure. The consequences of *Trim67* deletion on brain anatomy and behavior indicate that TRIM67 plays critical, yet undefined roles in brain development and function.

## Materials and Methods

### Animals

Mouse lines were on a C57BL/7 background and were housed and bred at the University of North Carolina with approval from the Institutional Animal Care and Use Committee. Timed pregnant females were obtained by placing male and female mice together overnight; the following day was designated as E0.5 if the female had a vaginal plug. We previously generated mice in which the first exon of *Trim9*, which encodes the RING and two BBox domains, was flanked by LoxP sites (Winkle et al., 2014). Exon 1 was removed from the germline with cytomegalovirus (CMV)-Cre, and all TRIM9 protein was lost. Since *Trim9* and *Trim67* have similar gene architectures, we generated a conditional *Trim67* allele using a similar strategy of flanking exon one and ∼200 bp upstream of the ATG start site by LoxP sites via homologous recombination. Mice carrying the *Trim67^fl^* allele were crossed with CMV-Cre mice to delete *Trim67* in the germline and generate *Trim67^−/−^* mice. After Cre-mediated excision of exon 1 of *Trim67*, the subsequent 16 ATGs encode out-of-frame transcripts. This excision was confirmed by PCR genotyping. Mice expressing Cre under the Nex promoter and mice carrying a Tau^loxP-STOP-loxP^-GFP allele were generous gifts from Dr. Klaus-Armin Nave (Max-Planck Institute, Göttingen, Germany), and Dr. Eva Anton (University of North Carolina at Chapel Hill), respectively.

### Reagents

Antibodies: TRIM67 rabbit polyclonal generated using murine TRIM67 recombinant protein aa45-73 [used at 1:1000 for immunoblot, 1:500 for immunohistochemistry (IHC)]; TRIM9 rabbit polyclonal antibody (used at 1:1000 for immunoblot; Winkle et al., 2014); mouse monoclonal against GAPDH [sc-166545, Santa Cruz Biotechnology (SCBT), used at 1:2500]; mouse monoclonal against myc tag (sc-40, SCBT, used at 1:1000); mouse monoclonal against HA tag (05-904, Millipore, used at 1:1000); and mouse monoclonal against β-III-tubulin (801202, BioLegend, used at 1:2000 for immunoblot, 1:1000 for IHC). Fluorescent secondary antibodies labeled with Alexa Fluor 568 or Alexa Fluor 647 were obtained from Invitrogen.

The plasmid encoding myc-tagged human TRIM9 aa139-781 (myc-TRIM9ΔRING) was described previously (Winkle et al., 2014). The plasmid encoding myc-tagged mouse TRIM67 aa158-783 (myc-TRIM67ΔRING) was generated by cloning the partial mouse TRIM67 sequence into the pcs2 vector. pcDNA3-DCC-HA (HA-DCC) was a generous gift from Dr. Marc Tessier Lavigne (Rockefeller).

### Cell culture and Western blotting

Embryonic day (E) 15.5 dissociated cortical neuron cultures were prepared as previously described (Viesselmann et al., 2011), and lysed at 2 d *in vitro* in modified RIPA buffer (50 mM Tris-HCl, pH 7.5, 200 mM NaCl, 0.5% NP-40, 2 mM MgCl_2_, 300 mM sucrose, 15 mM sodium pyrophosphate, 50 mM NaF, 40 mM β-glycerophosphate, 1 mM sodium vanadate, 150 µg/ml phenylmethanesulfonyl fluoride, 2 mM dithiothreitol, 5 mM N-ethylmaleimide, 3 mM iodoacetamide, 2 µg/mL leupeptin, and 5 µg/ml aprotinin). Protein from embryonic and adult tissues was obtained by dissection at E15.5 and postnatal day (P) 78, respectively, followed by lysis in modified RIPA buffer. SDS-PAGE and immunoblot analysis were performed using standard procedures with far-red-conjugated secondary antibodies (LI-COR Biosciences) imaged with an Odyssey Imager (LI-COR Biosciences).

### Immunoprecipitation

Coimmunoprecipitation assays were conducted according to standard procedures. Briefly, HEK293T cells were transfected using Lipofectamine 2000 (Invitrogen) by manufacturer protocol. Protein A/G beads (SCBT) coupled with a mouse anti-myc (9E10) antibody were incubated with lysates overnight at 4°C. Beads were washed three times with lysis buffer and bound proteins were resolved by standard SDS-PAGE and immunoblot analysis.

### Elevated plus maze

Mice were given one 5-min trial on the plus maze, which had two walled arms (closed arms, 20 cm in height) and two open arms. The maze was elevated 50 cm from the floor, and the arms were 30 cm long. Animals were placed on the center section (8 × 8 cm) and allowed to freely explore the maze. Measures were taken of time in, and number of entries into, the open and closed arms.

### Marble-bury test

Mice were tested in a Plexiglas cage located in a sound-attenuating chamber with ceiling light and fan. The cage contained 5 cm of corncob bedding, with 20 black glass marbles (14 mm in diameter) arranged in an equidistant 5 × 4 grid on top of the bedding. Subjects were given access to the marbles for 30 min. Measures were taken of the number of buried marbles (two thirds of the marble covered by the bedding).

### Olfactory test

Several days before the olfactory test, an unfamiliar food (Froot Loops, Kellogg’s) was placed overnight in the home cages of the mice. Observations of consumption were taken to ensure that the novel food was palatable. Sixteen to twenty hours before the test, all food was removed from the home cage. On the day of the test, each mouse was placed in a large, clean tub cage (46 cm long × 23.5 cm wide × 20 cm high), containing paper chip bedding (3 cm deep), and allowed to explore for 5 min. The animal was removed from the cage, and one Froot Loop was buried in the cage bedding. The animal was then returned to the cage and given 15 min to locate the buried food. Measures were taken of latency to find the food reward.

### Hotplate test

Individual mice were placed in a tall plastic cylinder located on a hotplate, with a surface heated to 55°C (IITC Life Science, Inc.). Reactions to the heated surface, including hindpaw lick, vocalization, or jumping, led to immediate removal from the hotplate. Measures were taken of latency to respond, with a maximum test length of 30 s.

### Open field assay

Mice were given a 1-h trial in an open field chamber (41 × 41 × 30 cm) crossed by a grid of photobeams (VersaMax system, AccuScan Instruments). Counts were taken of the number of photobeams broken during the trial in 5-min intervals, with separate measures for locomotion (total distance traveled) and rearing movements. Time spent in the center region of the open field was measured as an index of anxiety-like behavior.

### Acoustic startle and prepulse inhibition

Subjects were given two acoustic startle tests (SR-LAB system, San Diego Instruments), one at age 10–11 weeks and another at age 17–19 weeks. Mice were placed in a small Plexiglas cylinder within a larger, sound-attenuating chamber. The cylinder was seated on a piezoelectric transducer, which allowed vibrations to be quantified and displayed on a computer. The chamber included a ceiling light, fan, and a loudspeaker for the acoustic stimuli. Background sound levels (70 dB) and calibration of the acoustic stimuli were confirmed with a digital sound level meter (San Diego Instruments).

Each session consisted of 42 trials that began with a 5-min habituation period. There were seven different types of trials: the no-stimulus trials, trials with the acoustic startle stimulus (40 ms; 120 dB) alone, and trials in which a prepulse stimulus (20 ms; either 74, 78, 82, 87, or 90 dB) occurred 100 ms before the onset of the startle stimulus. Measures were taken of the startle amplitude for each trial across a 65-ms sampling window, and an overall analysis was performed for each subject’s data for levels of prepulse inhibition at each prepulse sound level (calculated as: 100 – [(startle response with prepulse/startle response with no prepulse) × 100]).

### Accelerating rotarod

In the first test session, mice were given three trials on an accelerating rotarod (Ugo Basile, Stoelting Co) with 45 s between each trial. Two additional trials were given 48 h later. Revolutions per minute was set at an initial value of three, with a progressive increase to a maximum of 30 across 5 min (the maximum trial length). Measures were taken for latency to fall from the top of the rotating barrel.

### Gait analysis

A track of Whatman filter paper (Sigma-Aldrich) was placed in a 7.5-cm-wide, 40-cm-long Plexiglas corridor closed at one end and open to a 10 × 10-cm Plexiglas chamber at the other end. The chamber was covered with an opaque cloth and filled with several pellets of dry food. Mouse forepaws and hindpaws were painted with two colors of Crayola Washable Kids’ Paint (Crayola, LLC) before the mouse was placed at the closed end of the Plexiglas corridor. Once mice had walked the length of the corridor, they were given 45 s in the recovery chamber before being returned to their home cage.

### Rolling wire-hang

Mice were held near a circular rubber gasket, suspended from a pulley, such that the mouse grasped the loop (Hoffman and Winder, 2016). Mice were released once all four paws were engaged, and latency to fall was recorded in three trials separated by 45 s each. Latency to fall was measured in seconds, and then multiplied by weight to produce hang impulse.

### Three-chamber sociability assay

This procedure consisted of three 10-min phases: a habituation period, a test for sociability, and a test for social novelty preference. For the sociability assay, mice were given a choice between being in the proximity of an unfamiliar, sex-matched C57BL/6J adult mouse (stranger one) versus being alone. In the social novelty phase, mice were given a choice between the already-investigated stranger one, versus a new unfamiliar mouse (stranger two). The social testing apparatus was a rectangular, three chambered box fabricated from clear Plexiglas. Dividing walls had doorways allowing access into each chamber. An automated image tracking system (Noldus Ethovision) provided measures of time spent within 5 cm of the Plexiglas cages (the cage proximity zone), and entries into each side of the social test box.

At the start of the test, the mouse was placed in the middle chamber and allowed to explore for 10 min, with the doorways into the two side chambers open. After the habituation period, the test mouse was enclosed in the center compartment of the social test box, and stranger one was placed in one of the side chambers. The stranger mouse was enclosed in a small Plexiglas cage drilled with holes, which allowed nose contact. An identical empty Plexiglas cage was placed in the opposite side chamber. Following the placement of the stranger and the empty cage, the doors were reopened, and the subject was allowed to explore the social test box for a 10-min session. At the end of the sociability phase, stranger two was placed in the empty Plexiglas container, and the test mouse was given an additional 10 min to explore the social test box.

### Morris water maze

The water maze consisted of a large circular pool (diameter = 122 cm) partially filled with water (45 cm deep, 24–26°C), located in a room with numerous visual cues. The procedure involved three different phases: a visible platform test, acquisition in the hidden platform task, and reversal learning. In the visible platform test, each mouse was given four trials per day, across 2 d, to swim to an escape platform cued by a patterned cylinder extending above the surface of the water. For each trial, the mouse was placed in the pool at one of four possible locations (randomly ordered), and then given 60 s to find the visible platform. If the mouse found the platform, the trial ended, and the animal was allowed to remain 10 s on the platform before the next trial began. If the platform was not found, the mouse was placed on the platform for 10 s, and then given the next trial. Measures were taken of latency to find the platform and swimming speed via an automated tracking system (Noldus Ethovision).

For the hidden platform task, the platform (diameter = 12 cm) was submerged and the water made opaque using white Crayola Washable Kids’ Paint (Crayola, LLC). Each animal was given four trials per day, with 1 min per trial, to swim to the hidden platform. Criterion for learning was an average group latency of 15 s or less to locate the platform. Mice were tested until the group reached criterion, with a maximum of 9 d of testing. When criterion was reached, mice were given a one min probe trial in the pool with the platform removed. Selective quadrant search was evaluated by measuring percentage time spent in the target quadrant and number of crossings over the location where the platform (the target) had been placed during training, versus the corresponding area in the opposite quadrant. During the subsequent reversal learning phase, the escape platform was placed in a different quadrant, and mice were tested until the group reached the learning criterion, using the same procedure as with initial acquisition. A second one min probe trial was conducted at the end of the reversal learning phase.

### Brain fixation and sectioning

All adult mice used for neuroanatomical studies were anesthetized with an intraperitoneal injection of 1.2% avertin and intracardially perfused with 1× PBS followed by 4% paraformaldehyde (PFA). Brains were removed and fixed in 4% PFA for an additional 48 h, rinsed with 1× PBS, and washed in 70% EtOH for at least 24 h before vibratome sectioning at 100-µm thickness. Embryonic brains were removed at E15.5 and drop-fixed in 4% PFA for 72 h, rinsed with 1× PBS, and embedded in 1% agarose before cryoprotection with 30% sucrose and cryostat sectioning at 20-µm thickness.

### IHC and immunocytochemistry

Sections were blocked in 0.2% Triton X-100 and 10% BSA in 1× PBS for 2 h on a rocker at room temperature. Solution was replaced with primary antibodies in blocking solution overnight at 4°C. Sections were washed three times for 20 min each in 1× PBS, and then incubated with secondary antibodies in blocking solution for two h at room temperature. Sections were then incubated with 300 nM DAPI in 1× PBS for 30 min and then washed three times for 20 min each with 1× PBS. Sections were mounted in mounting media (20 mM Tris, pH 8.0, 90% glycerol, and 0.5% N-propyl gallate) and sealed with nail polish. Fluorescently labeled sections were imaged on a Zeiss LSM 780 confocal microscope using a 20× Plan-Apochromat objective. Embryonic cortical neurons cultured on nitric-acid-washed coverslips and fixed with 4% PFA were stained by the same protocol and imaged on an Olympus IX81-ZDC2 inverted microscope using a 100 × 1.49 NA UAPON DIC/TIRF objective.

### Histologic measurements

Five-week-old brains from an equal number of male and female mice from both genotypes were sectioned and stained with Black Gold II (Histo-Chem, Inc.) per manufacturer protocol, mounted in DPX mounting media (VWR International), and imaged on a Leica WILD M420 macroscope using an APOZOOM lens. Sections were aligned to bregma using a reference atlas, and individual regions were outlined as described below and measured using ImageJ. White matter in the striatum was defined using the Trainable Weka Segmentation v3.2.2 plugin for ImageJ (Arganda-Carreras et al., 2017) trained on two classes (white and gray matter) with at least eight examples per class. Brain regions and fiber tracts were measured in each section, then individually aligned by minimizing the total standard deviation, as the shapes of regions along the anterior-posterior axis were not apparently different between individual animals.

All structures present in each hemisphere (hippocampus, amygdala, CPu, etc.) were reported as the average size of the structure in one hemisphere. Fiber tracts were demarcated by myelin staining. The thicknesses of the corpus callosum, hippocampal commissure and anterior commissure were measured at the midline perpendicular to the direction of the tract. The cortical thickness was determined by measuring the area of a cortical region divided by the average of the lengths of the cingulum and external surfaces to produce an average thickness along the length of the cortex. Hippocampal area was defined as the area bounded by the fimbria, thalamus, habenula, third ventricle, and corpus callosum, which included both the dentate gyrus and Ammon’s horn. To measure the area of the lateral and basolateral amygdala, which are bounded by the external and amygdalar capsules, boundaries along these capsules were drawn on black gold II myelin stained sections and were connected with a straight perpendicular line at the medial inferior ends. The striatum was bounded by the lateral ventricles, and black gold stained corpus callosum, external capsule, and anterior commissure in anterior sections, and the lateral ventricles, corpus callosum, external capsule, globus pallidus, and internal capsule in posterior sections. The thalamus was delineated by the habenulae, hippocampus, fimbriae, internal capsules, and a straight line drawn between the inferior tip of the internal capsules. Anatomic regions were compared to reference atlases to confirm boundaries (Franklin and Paxinos, 2008; Allen Mouse Brain Atlas).

### Protein sequence comparison

Protein sequences for Class I TRIM proteins were retrieved from the UniprotKB/Swiss-Prot (accession numbers: Q9C026-1, Q8C7M3-3, Q6NZX0, F7E188, A0A1D5Q2P9, Q91ZY8, A0A1D5PZ60, M9MRI4, B1GRL4, Q6ZTA4-3, Q505D9-1, A0A286YAS5, F6U581, F6S2G3, D3ZTX1, A0A1L8F061, A0A1L8G7R8, O15344, Q9NQ86, Q9UJV3, Q7Z4K8, O70583, Q7TNM2, Q80WG7, Q9QUS6, E7FAU0, E7F5U6, F1QUB4, F6P3Q3, E7F8R6, F7A2I4, Q6DEU6, F1NE64, Q90WD1, A0A0G2JXN2, A0A0G2JW11, A0A0G2JWJ3, P82458, D8WX03, Q6NU77, T2M630, A0A0V1B9S1, A0A087ZYT3, A0A195FV05, A0A0P5WD37, I3LLR6, A0A287BHA8, F1RLE9, F1RGR8, K7GSV3, F1SG17, U3JIW9, U3JJW0, U3JGS8, U3JPI3, U3KBH9, E2RAN1, E2QZT5, F6V5Z8, E2RKC8, F6V1Y9, E2R7N3, A0A1S3SS87, A0A1S3S8H3, A0A1S3PJZ7, A0A1S3M2Y3, A0A1S3NWR8, A0A1S3RQU3, A7S501, A7S7Q5), NCBI GenPept (accession numbers: XP_015139864.1, EKC37550.1, XP_017952420.1, XP_015133862, XP_005099658.1, XP_005110236.1, XP_012560769.1, XP_014452196.1, XP_014463268.1, XP_014465414.1, XP_006266936.2, XP_006030994.1, XP_019349655.1, XP_005043498.1, XP_014770719.1, XP_014769144.1, KXJ14478.1, XP_020900170.1, XP_020618149, XP_020618148.1, XP_009012087.1, XP_009163316.1, XP_009162620.1, XP_002117207.1, XP_002741117.1, XP_006818271.1, XP_006823565.1, XP_022109997.1, XP_022104238.1), and EnsemblMetazoa (accession number CapteT155810) databases. Sequences were compared using the ClustalX2 multiple sequence alignment, with default parameters and three full alignment iterations (Sievers et al., 2011). Phylogenetic trees were generated using the nearest neighbor joining algorithm of ClustalX2 with 1000 bootstrapping iterations and organized with FigTree v1.4.3.

### Statistical methods

Behavioral data were analyzed using one-way or repeated measures ANOVA, with factor genotype. Separate ANOVAs for each sex were conducted for measures of body weight. Fisher’s protected least-significant difference (PLSD) tests were used for comparing group means only when a significant *F* value was determined. Within-group comparisons were conducted to determine side preference in the three-chamber test for social approach and for quadrant preference in the Morris water maze.

For all brain regions measured in multiple sections, comparisons were performed by two-way ANOVA. Sections and genotypes were treated as groups with each brain a subject, with genotype as the tested factor. For the paired brain weight and Nex-Cre:*Trim67^fl/fl^* strain corpus callosum comparisons, Wilcoxon signed-rank tests were used. For unpaired single measurements, Mann–Whitney pairwise comparisons were made. A χ^2^ test was used to compare the heterozygote birth rate to the predicted Mendelian distribution. For all comparisons, α = 0.05.

## Results

### Phylogenetic analysis of Class I TRIMs reveals evolutionary conservation of TRIM67

**Figure 1.**
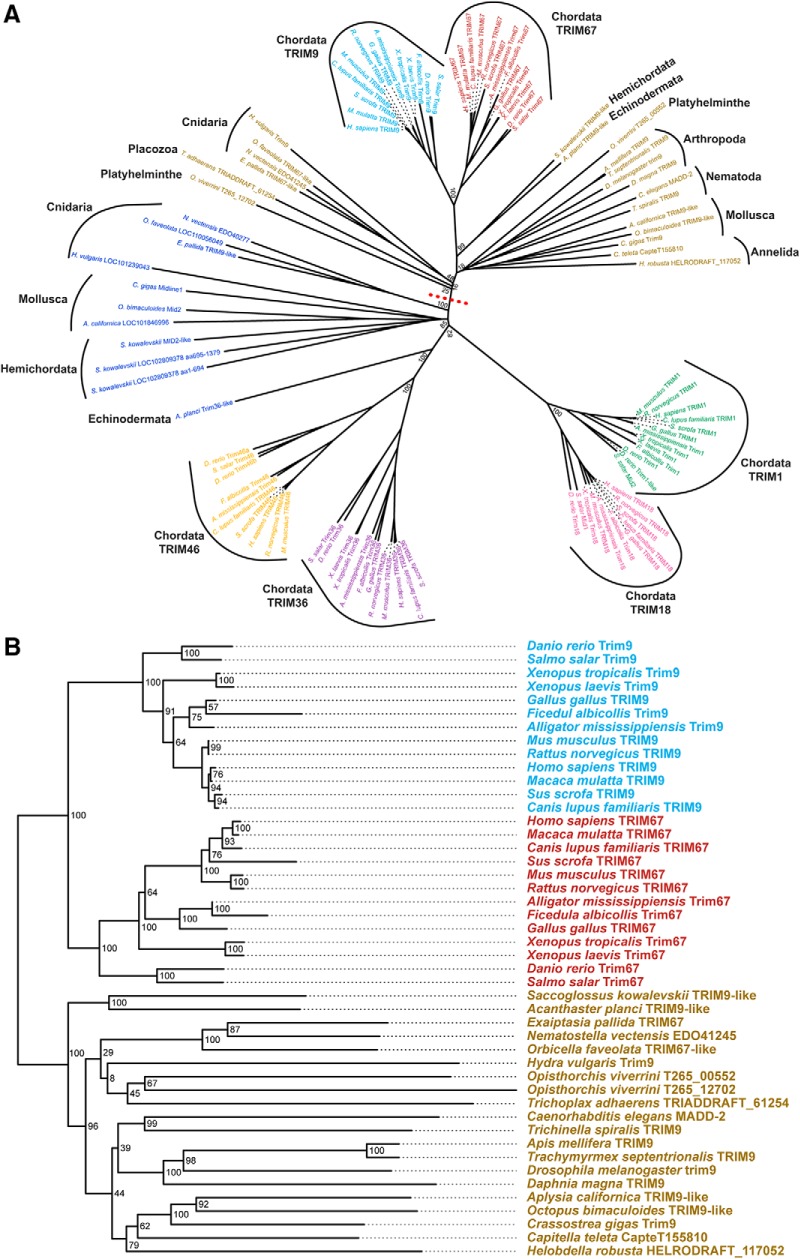
TRIM67 is evolutionarily conserved. ***A***, Phylogeny of Class I TRIM proteins across animal phyla based on protein sequences, showing the six members in vertebrate species (cyan, TRIM9; red, TRIM67; green, TRIM1/MID2; pink, TRIM18/MID1; purple, TRIM36; orange, TRIM46) and the one to two members in nonvertebrate animal phyla (blue, TRIM1/18/36/46-like; brown, TRIM9/67-like). Dashed red line separates TRIM9/67-like proteins from TRIM1/18/36/46-like proteins. Bootstrap values are shown at major group branches. ***B***, Phylogenetic tree of invertebrate TRIM9/67-like proteins alongside several vertebrate TRIM9 and TRIM67 homologs (all proteins above dashed red line in ***A***). Vertebrate TRIM67 exhibits higher sequence similarity than TRIM9 to invertebrate Class I TRIMs. Numbers denote bootstrapping values.

Comparisons of human Class I TRIMs to *D. melanogaster* trim9 suggested that human TRIM9 was the most evolutionarily conserved (Short and Cox, 2006; Morikawa et al., 2011). In light of the divergence in phenotypes associated with the loss-of-function mutations in invertebrate trim9 compared to deletion of murine *Trim9* (Hao et al., 2010; Morikawa et al., 2011; Winkle et al., 2014; Menon et al., 2015), we compared the sequences of Class I TRIMs from a variety animal phyla, which revealed interesting features of this family. Based on current genome annotations, no Class I TRIMs were found in more simple animal clades such as Choanoflagellata, Porifera, or Ctenophora but were present in Placozoa, Cnidaria, and phyla within the Bilateria. TRIM67 and TRIM9 were the most conserved vertebrate Class I TRIMs, as determined by branch length from the sole Class I TRIM in Placozoa to each vertebrate ortholog pair [Fig. 1*A*, TRIM9/67: 0.4866; TRIM46/36: 0.5213; TRIM1/18: 0.6253 substitutions per site (sps)]. Cnidaria and several Bilaterian clades including Mollusca, Echinodermata, Hemichordata, and Platyhelminthe had more than one Class I TRIM, indicating a gene gain event. One of these was closest to TRIM9/67 (Fig. 1*A*, average distance to TRIM9/67 pair = 0.4098 sps; average distance to TRIM1/18 and TRIM46/36 pairs = 0.5368 sps), the other was closer to the chordata TRIM46/36 and TRIM1/18 pairs (Fig. 1*A*, average distance to TRIM9/67 pair = 0.5964 sps; average distance to TRIM1/18 and TRIM46/36 pairs = 0.58127 sps). Interestingly, this second Class I TRIM was absent from a subset of Bilaterian clades, including Arthropoda, Nematoda, and Annelida, suggesting a subsequent gene loss event. This broader phylogenetic analysis indicated that Chordata TRIM67 homologs exhibited higher sequence similarity to ancestral Class I TRIMs, and the single Class I TRIM present in *Drosophila* and *C. elegans,* suggesting vertebrate TRIM67 homologs were more evolutionarily conserved than vertebrate TRIM9 homologs (Fig. 1*B*, TRIM67: 0.1155; TRIM9: 0.1478 sps, from nearest non-vertebrate branch).

### Generation of *Trim67^−/−^* mice

**Figure 2.**
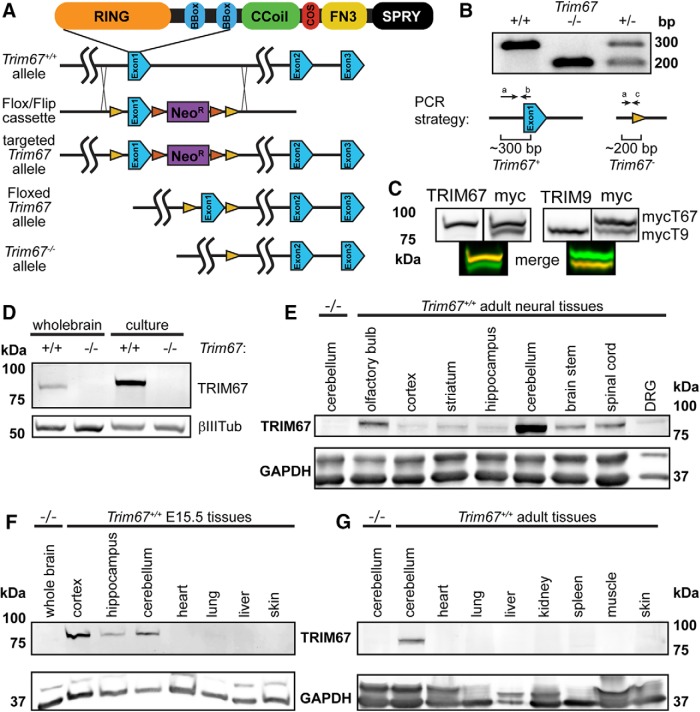
Generation of *Trim67*^−/−^ mouse and TRIM67 brain localization. ***A***, Diagram of targeting strategy for the *Trim67* gene showing Cre-lox mediated excision of exon 1. This excision leads to the next 16 intronic and 4 exonic ATG codons being out of frame. LoxP sites are yellow, FLIP sites are orange, and neomycin resistance gene (NeoR) is purple. ***B***, Agarose gel separation of genotyping PCR products, demonstrating deletion of both copies of the first exon of *Trim67* (-/-) or one copy in a heterozygote (±). Diagrams show PCR products from wild-type and knock-out alleles. ***C***, Duplicate Western blottings of lysate from HEK293T cells expressing both myc-TRIM9 and myc-TRIM67, were probed with both the indicated TRIM antibody (left, red) and myc (right, green), and spectrally distinct secondary antibodies. The newly generated TRIM67 polyclonal antibody recognizes TRIM67 but not TRIM9; the polyclonal TRIM9 antibody recognizes TRIM9, but not TRIM67, as can be seen in the merged images below. ***D***, Western blotting of whole-brain or 2 d *in vitro* dissociated cortical neuron lysate from *Trim67^+/+^* and *Trim67^−/−^* E15.5 embryos probed for TRIM67 and βIII-tubulin as a loading control. ***E***–***G***, Western blotting detects TRIM67 expression in various adult (***E***) and embryonic (***F***) neural tissues, but not in the examined tissues outside the nervous system (***G***). GAPDH is a loading control. Age-matched *Trim67^−/−^* lysate from indicated tissue shown in left most lane of each blot.

**Figure 3.**
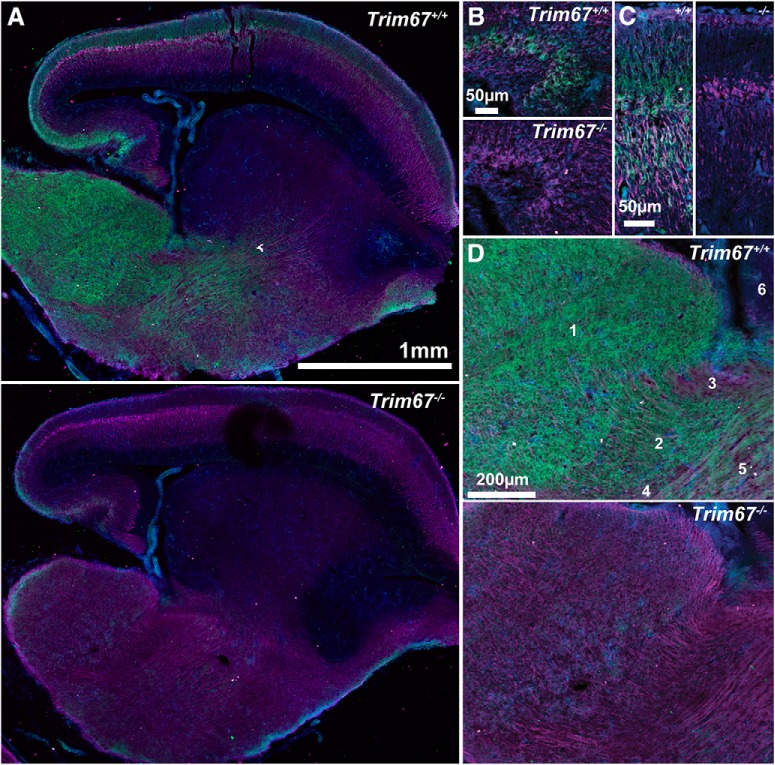
TRIM67 is present in multiple murine brain regions. ***A***, Low-magnification sagittal sections of *Trim67^+/+^* and *Trim67^−/−^* E15.5 brains stained for TRIM67 (green), β-III-tubulin (magenta), and nuclei (DAPI, blue). TRIM67 is present in cell bodies in the developing hippocampus (***B***) and cortex (***C***) at E15.5. ***D***, TRIM67 expression is evident in the peduncular hypothalamus (5), diencephalon (1), and reticular complex (2), but not in the prethalamic eminence (3), zona incerta complex (4), or subpallium (6).

Based on the evolutionary conservation of Class I TRIM proteins, and the established, independent roles of the other five Class I TRIMs in the developing nervous system, we sought to investigate how loss of TRIM67 affected brain development. We generated a conditional murine *Trim67* allele (Fig. 2*A*, *T**rim67^fl^*). Mice carrying the *Trim67^fl^* allele were crossed with CMV-Cre mice to delete *Trim67* in the germline and generate *Trim67^−/−^* mice, confirmed by multiplexed PCR genotyping (Fig. 2*B*). *Trim67^−/−^* mice were viable, and heterozygous adults produced the expected Mendelian ratio of offspring (^+/+^ 102/87; ± 157/174; *^−/−^* 89/87; χ^2^ = 0.117). We developed specific polyclonal antibodies to TRIM9 and TRIM67 using unique regions within their respective N termini (Fig. 2*C*). Western blotting of lysate from embryonic whole-brain and dissociated cortical neuron culture at 2 d *in vitro* using the TRIM67 antibody confirmed loss of TRIM67 protein (Fig. 2*D*). TRIM67 was detected in most sampled regions of the adult and embryonic nervous systems, including dorsal root ganglion in the adult peripheral nervous system, but it was not detected in the tested non-neuronal tissues (Fig. 2*E–G*). Of brain regions tested, TRIM67 protein was most enriched in the embryonic cortex and the adult cerebellum. Immunohistochemical staining of sagittal sections of embryonic brains (Fig. 3*A*) demonstrated high TRIM67 expression throughout the developing hippocampus (Fig. 3*B*), the cortex (Fig. 3*C*), and most structures of the nascent diencephalon (Fig. 3*D*). This staining was absent in *Trim67^−/−^* embryonic brains (Fig. 3). Unfortunately, our TRIM67 antibody exhibited nonspecific staining in adult tissue by IHC, hindering examination of TRIM67 localization in the mature brain.

### TRIM67 interacts with TRIM9 and DCC

**Figure 4.**
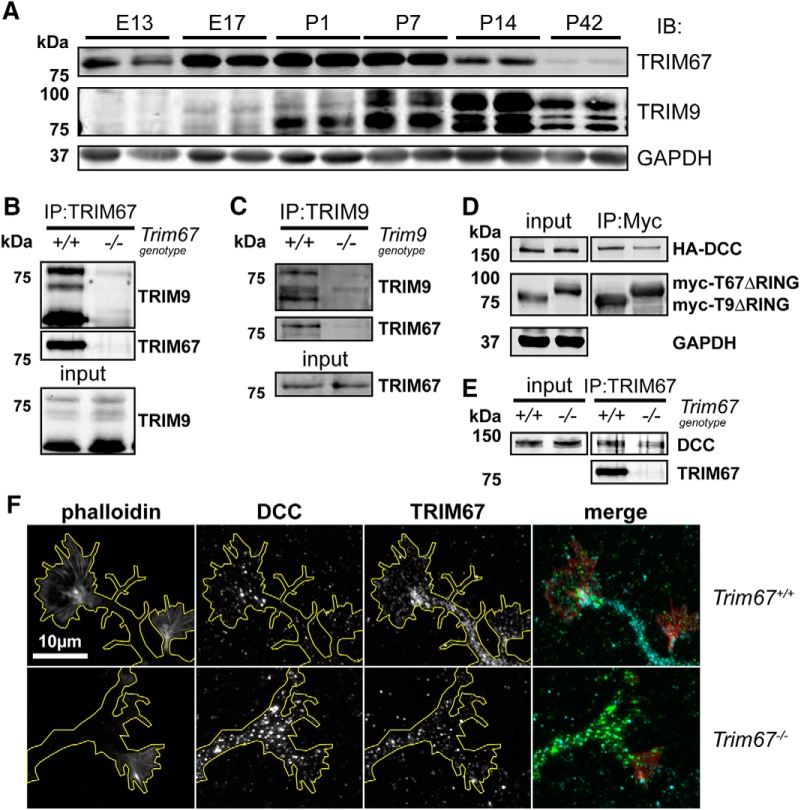
TRIM67 is expressed in developing cortex and interacts with TRIM9 and DCC. ***A***, Western blottings of cortical lysate from indicated embryonic (E) and postnatal (P) ages reveal expression patterns of TRIM67 and the three isoforms of TRIM9. ***B***, Western blotting of TRIM67 immunoprecipitates from *Trim67^+/+^ and Trim67^−/−^*mouse cortical neuronal lysate probed for TRIM9 and TRIM67. Coimmunoprecipitation of TRIM9 occurred in the presence of TRIM67. ***C***, A similar immunoprecipitation of TRIM9 from embryonic brain lysates coimmunoprecipitates TRIM67 in wild-type cortical lysate, but not in the absence of TRIM9. ***D***, HEK293T cells were cotransfected with HA-tagged DCC and either myc-tagged TRIM9 or TRIM67 lacking the RING domain (ΔRING). Immunoprecipitation of either myc-TRIMΔRING coprecipitated HA-DCC. ***E***, The top of the blot shown in ***B***, probed for DCC, showing that endogenous DCC is enriched over background levels in TRIM67 immunoprecipitates from wild-type embryonic brain lysate. The TRIM67 IP is the same as in ***B***. ***F***, Photomicrographs of neurites and growth cones from *Trim67^+/+^* and *Trim67^−/−^* cultured embryonic cortical neurons show DCC immunostaining in the same cells as TRIM67. Residual TRIM67 staining in *Trim67^−/−^* is due to nonspecific binding of the antibody. In merged image, phalloidin is red, DCC is green, and TRIM67 is cyan.

Using our newly developed TRIM67 and TRIM9 antibodies, we investigated protein levels of TRIM9 and TRIM67 in the developing cortex. We found that both proteins were present in the murine cortex at a range of ages. TRIM67 protein levels peaked late embryonically and perinatally, whereas levels of TRIM9 peaked later in development (Fig. 4*A*). TRIM proteins often heterodimerize, including Class I members TRIM1 and TRIM18 (Hatakeyama, 2011). We exploited coimmunoprecipitation assays to determine whether TRIM9 and TRIM67 could potentially heterodimerize. Using the TRIM67 antibody, endogenous TRIM9 was coimmunoprecipitated from wild-type embryonic cortical lysate but not from *Trim67^−/−^* lysate (Fig. 4*B*). In the reciprocal assay, the TRIM9 antibody coimmunoprecipitated endogenous TRIM67 from wild-type embryonic cortical lysate, but not from *Trim9^−/−^* lysate (Fig. 4*C*). These data indicate TRIM9 and TRIM67 interact and may heterodimerize.

Mammalian TRIM9 and the Class I TRIM in *D. melanogaster* and *C. elegans* interact with the netrin-1 receptor DCC (or its invertebrate ortholog) and regulate netrin-dependent axonal responses (Hao et al., 2010; Morikawa et al., 2011; Winkle et al., 2014). In light of the enriched expression of TRIM67 in the developing cortex, the diverse phenotypes associated with loss of mammalian TRIM9 and the invertebrate trim, and the evolutionary conservation of TRIM67, we hypothesized that TRIM67 may also interact with DCC and represent a more functional homolog to the invertebrate Class I TRIM. Coimmunoprecipitation of HA-tagged DCC occurred with either myc-tagged TRIM67ΔRING or myc-TRIM9ΔRING from HEK293T lysates, demonstrating that both mammalian TRIM9 and TRIM67 were capable of interacting with DCC (Fig. 4*D*). These constructs were used as deletion of the RING domain (ΔRING) stabilizes transient interactions between TRIMs and their interacting partners (Kim et al., 2015). Endogenous DCC was also enriched over background in endogenous TRIM67 immunoprecipitates from embryonic brain lysate (Fig. 4*E*). Immunocytochemistry also revealed that TRIM67 and DCC were present simultaneously in dissociated embryonic cortical neurons (Fig. 4*F*). Together these data indicate that TRIM67 interacts with both TRIM9 and DCC.

### Cortical commissural fiber tracts

**Figure 5.**
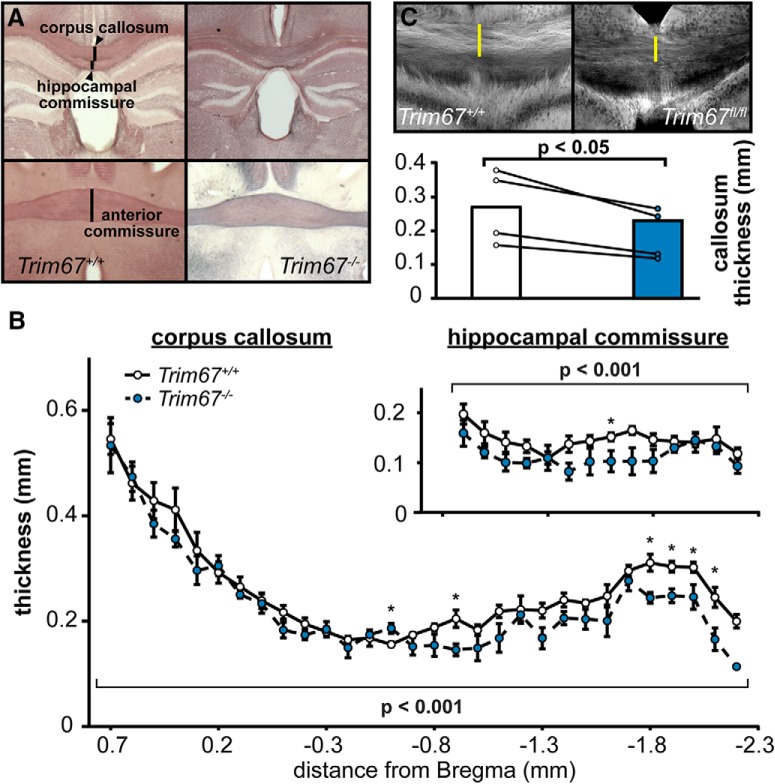
*Trim67* deletion reduces the thickness of certain fiber tracts. ***A***, Photomicrographs of black-gold stained coronal brain sections, the fiber tracts measured are indicated. ***B***, The corpus callosum was thinner in *Trim67^−/−^* mice (*p* = 0.00000909), with most significant individual measures toward the caudal end of the tract. The hippocampal commissure, including both the fornix and dorsal hippocampal commissure, was reduced in thickness in *Trim67^−/−^* mice (*p* = 0.000215). ***C***, GFP staining in the corpus callosum of Nex-Cre:Tau^lox-STOP-lox^-GFP:*Trim67^+/+^* and Nex-Cre:Tau^lox-STOP-lox^-GFP:*Trim67^fl/fl^* brains. The corpus callosum was thinner (*p* = 0.0304) in *Trim67^fl/fl^* mice (*p < 0.05 at this position).

All these findings prompted us to investigate netrin-dependent fiber tracts in *Trim67^−/−^* mice. Loss of murine *Ntn1* or *Dcc* leads to a narrower anterior commissure and agenesis of the corpus callosum and hippocampal commissure (Serafini et al., 1996; Fazeli et al., 1997). We measured the size of these axon tracts in 5 adult *Trim67^+/+^*(1 female, 4 males) and 4 adult *Trim67^−/−^* (two females, two males) littermate-paired brains, sectioned and stained for myelin (Fig. 5*A*). Deletion of *Trim67* had no effect on the size of the anterior commissure (*Trim67^+/+^*: 0.495 ± 0.023 mm; *Trim67^−/−^*: 0.493 ± 0.013 mm; *p* = 0.944, Mann–Whitney) but did decrease the thickness of the corpus callosum (Fig. 5*B*). Additionally, the hippocampal commissure, measured as the combined dorsal fornix and dorsal hippocampal commissure, was thinner in *Trim67^−/−^* mice (Fig. 5*B*, inset). Taken together, these data suggest that loss of *Trim67* decreases the size of cortical and hippocampal axon tracts, but not all commissures. To better determine whether differences in the corpus callosum were due to loss of *Trim67* from cortical neurons, we crossed *Trim67^fl/fl^* with mice expressing Cre under the Nex promoter (Goebbels et al., 2006) and Tau-^lox-STOP-lox-GFP^ mice (Higginbotham et al., 2012), resulting in *Trim67* deletion and GFP expression in the neocortex and hippocampus. The corpus callosum thickness of *NexCre/Trim67^fl/fl^*/Tau-^lox-STOP-lox-GFP^ mice (two females, two males) was also reduced when compared to *NexCre/Trim67^+/+^*/Tau-^lox-STOP-lox-GFP^ littermates (two females, two males; Fig. 5*C*, *p* = 0.0304, Mann–Whitney). The intensity of GFP in the hippocampal commissure of these mice was insufficient to permit quantification of the thickness of this tract.

### Altered subcortical anatomy in *Trim67^−/−^* brains

**Figure 6.**
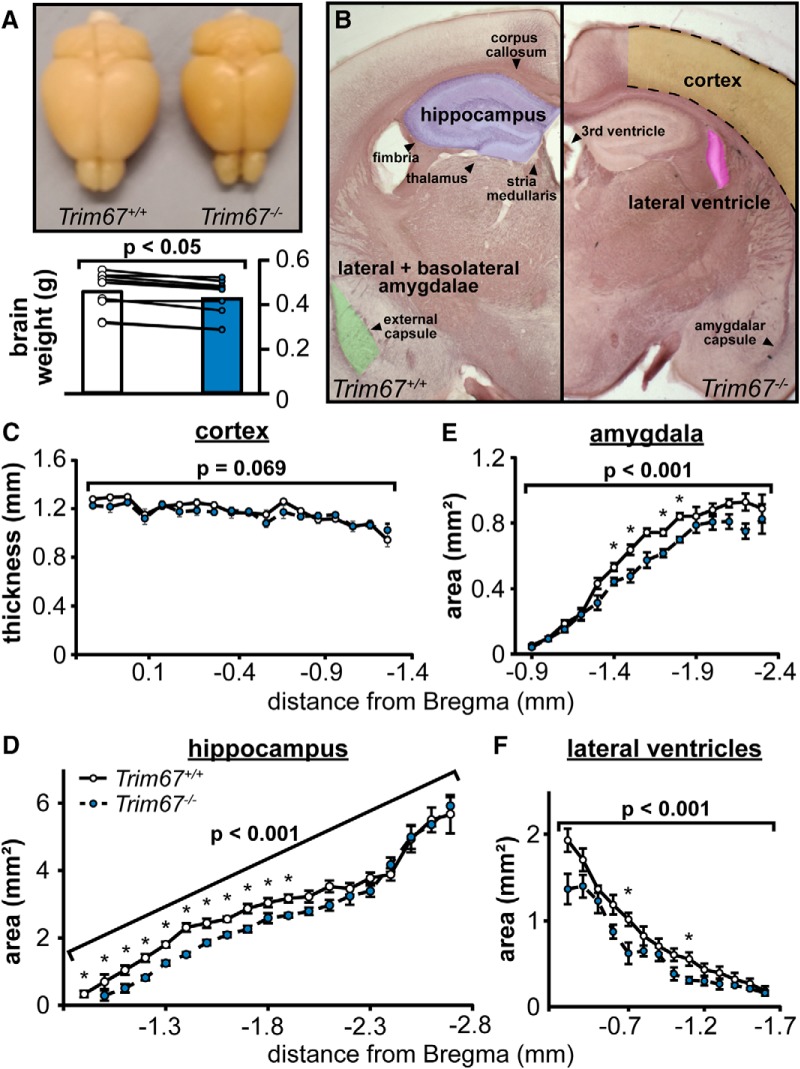
Reduction in total brain weight and hypotrophy of multiple brain areas occurs with loss of *Trim67.* ***A***, Photograph of representative *Trim67^+/+^* (left) and *Trim67^−/−^* (right) brains from five-week-old mice. Total weight of *Trim67^−/−^* brains was reduced by ∼10% when compared to littermate controls (*p* = 0.0108, Wilcoxon signed-rank test). ***B***, Photomicrographs of black-gold stained coronal sections of *Trim67^+/+^* and *Trim67^−/−^* brains at 1.3 mm posterior to bregma. Outlines delineate regions used for measures in this figure. ***C***, Deletion of *Trim67* had no effect on the thickness of the cortex (*p* = 0.069), measured from the cingulum to the cortical surface (average distance between the dashed black lines in ***B***). ***D***, Area of the hippocampal gray matter (including both the dentate gyrus and Ammon’s horn), reported as the average of both individual hemispheres from its first emergence through the fimbriae to 2.7 mm posterior to bregma. The hippocampus was smaller in *Trim67^−/−^* brains (*p* = 0.00000889), with significant individual measures toward the rostral portion. ***E***, Area of the amygdala, reported as the average of both individual hemispheres. The amygdala was significantly smaller in *Trim67^−/−^* brains (*p* = 0.000110). ***F***, Area of the lateral ventricles reported as the average of both individual hemispheres, from the first section posterior to the anterior commissure to the last section with visible ventricle. *Trim67^−/−^* lateral ventricles were smaller (*p* = 0.000128; *p < 0.05 at this position).

In investigating the effect of *Trim67* deletion on fiber tracts, we noted that *Trim67^−/−^* brains appeared smaller than *Trim67^+/+^* counterparts; postfixation weight of *Trim67^−/−^* brains was indeed ∼10% less than littermate counterparts (Fig. 6*A*). In light of this decrease, we hypothesized that fiber tract thinning may be due to general hypotrophy of the cortex and hippocampus, and therefore measured the sizes of these brain regions in the same sections (Fig. 6*B*). However, there was no difference in the thickness of the cortex between *Trim67^+/+^* and *Trim67^−/−^* brains (Fig. 6*C*), but the hippocampus was smaller in the absence of *Trim67* (Fig. 6*D*). Further, the anterior aspect of the hippocampal gray matter began ∼100 µm posterior in *Trim67^−/−^* mice compared to *Trim67^+/+^* mice, when aligned anatomically (Fig. 6*D*, bregma -1.0 and -0.9, respectively). We also observed that the amygdala (Fig. 6*E*) and lateral ventricles (Fig. 6*F*) were smaller in *Trim67^−/−^* brains.

### Behavioral phenotyping of *Trim67^−/−^* mice

**Figure 7.**
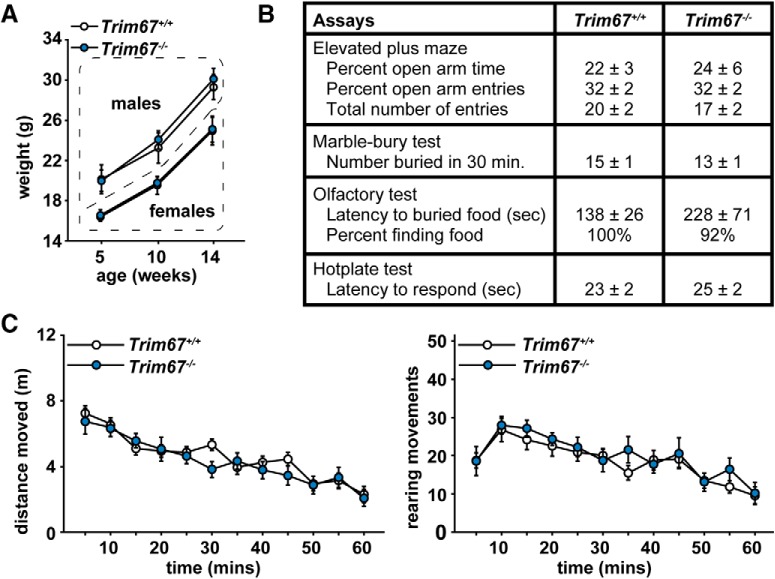
Mouse growth, sensory ability and general locomotion are not affected by *Trim67* deletion. ***A***, Weights of mice over the course of behavioral assays, showing no effect of *Trim67* loss on overall growth. ***B***, Results of elevated plus maze and several sensory assays. Units are indicated. ***C***, Locomotor results in the open field assay. None of these assays show significant results by ANOVA.

With the neuroanatomical defects in *Trim67^−/−^* mice and a wide range of functions of Class I TRIMs in neurons, we employed a comprehensive battery of behavioral assays to determine the ramifications of loss of *Trim67 in vivo*. The behavioral testing regimen (Table 1) has been standardized across multiple mouse strains (Moy et al., 2007, 2008, 2009, 2012; Huang et al., 2013; Nagy et al., 2017). For these studies, subjects were 15 *Trim67^+/+^* mice (seven males and eight females) and 13 *Trim67^−/−^* mice (seven males and six females) aged six weeks at the start of behavioral testing. Over the course of the behavioral testing, there was no significant effect of loss of *Trim67* on body weight or overall growth in either male or female mice (Fig. 7*A*). There were also no effects of genotype on anxiety-like behavior in an elevated plus maze, exploratory and perseverative digging in a marble-bury assay, olfactory ability in finding buried food, or thermal sensitivity (Fig. 7*B*). In an open field assay, loss of *Trim67* had no effect on locomotor activity or rearing movements (Fig. 7*C*) or in time spent in the center region of the field (*Trim67^+/+^* = 234 ± 47 s, *Trim67^−/−^* = 213 ± 32 s), a measure of anxiety. Normal behaviors in these tests indicate loss of *Trim67* does not detectably impact sensory abilities, general locomotion, or anxiety behaviors.

**Table 1.** Timeline of behavioral phenotyping assays

Age (weeks)	Procedure
6–7	Elevated plus maze test for anxiety-like behavior
7–8	Activity in a 1-h open field test
8–9	Rotarod test for motor coordination and motor learning
9–10	Social approach in a three-chamber choice task
10–11	Marble-bury assay for digging responses; prepulse inhibition of acoustic startle responses (first test)
11–12	Buried food test for olfactory ability
12–17	Morris water maze test for spatial learning
17–19	Prepulse inhibition of acoustic startle responses (second test); hot-plate test for thermal sensitivity

### Impaired spatial memory and cognitive flexibility

Changes in the size of the hippocampus and lateral ventricles, as observed in *Trim67^−/−^* brains, have been associated with spatial learning and memory deficits (Wright et al., 2000; Gaser et al., 2004; Boyer et al., 2007). The Morris water maze was used to assess spatial and reversal learning, swimming ability, and visual function. The procedure was divided into three phases: a visible platform test, acquisition with a hidden platform, and reversal learning. In the visible platform trials both groups showed similar times to escape, as well as similar swimming speeds (Table 2), indicating visual function was not affected by loss of *Trim67*.

**Table 2.** Morris Water Maze latencies and swim speeds

	*Trim 67* ^+/+^	*Trim 67* ^−/−^
Visible platform test		
Latency to platform (sec)		
Day 1	26 ± 3	24 ± 4
Day 2	9 ± 1	13 ± 2
Swim speed (cm/s) on day 1 of testing		
Visible platform test	16 ± 0.7	15 ± 0.8
Acquisition with hidden platform	17 ± 0.8	15 ± 1.1
Reversal learning	18 ± 0.6	14 ± 1.3*

**Figure 8.**
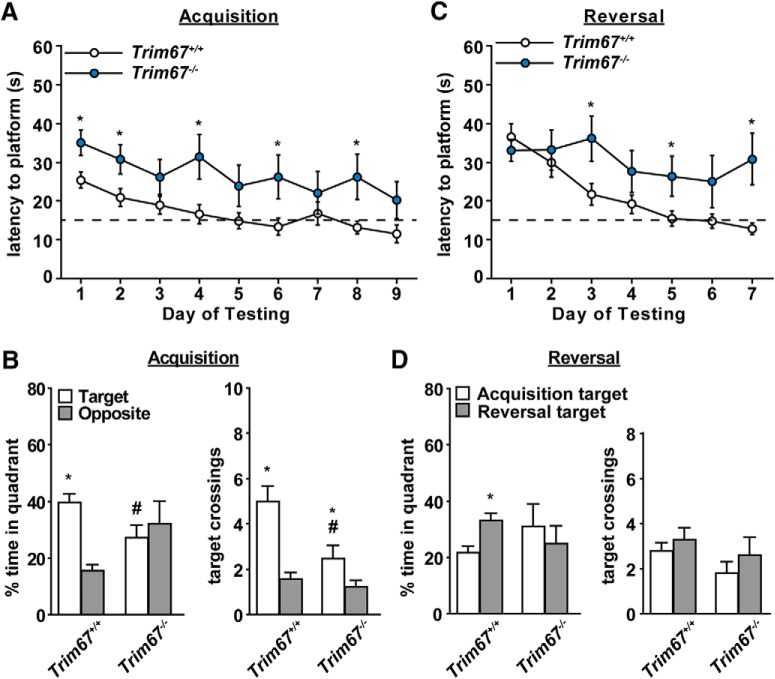
Loss of *Trim67* leads to impairments in spatial learning and memory. ***A***, Time (s) for mice to find a hidden platform in the Morris water maze, with a threshold for learning set at a group average of 15 s (dashed line). *Trim67^−/−^* mice fail to reach this threshold out to the maximum of 9 d of training and have overall significantly higher latencies to find the platform (*p < 0.05; all trials *p* = 0.0225). ***B***, Time spent in target and opposite quadrants and number of times crossing the previous platform position in a probe trial following acquisition day 9. Whereas *Trim67^+/+^* mice spent a higher amount of time in the quadrant previously occupied by the platform (*p = 0.0003), *Trim67^−/−^* mice failed to show this preference and spent a lower time in the target quadrant than *Trim67^+/+^* mice (#*p* = 0.0343). ***C***, Subsequent reversal trials demonstrated an increased latency to find the platform in the *Trim67^−/−^* cohort compared to *Trim67^+/+^* littermates (*p < 0.05; all trials *p* = 0.0149). ***D***, In a probe trial following reversal day 7, only *Trim67^+/+^* mice spent more time in the new target quadrant (*p = 0.0335).

Following the visible platform task, mice were evaluated for their ability to find a hidden escape platform submerged in opaque water. On the first day of hidden platform acquisition both groups again had similar swimming speeds, however *Trim67^−/−^* mice swam more slowly on the first day of the reversal learning test (genotype × testing phase interaction, *p* = 0.0256). *Trim67^−/−^* mice took significantly longer to find the hidden platform, and never reached the criterion for learning (Fig. 8*A*, dashed line, 15 s) by day 9 of testing. On trial day 9, two *Trim67^−/−^* mice failed to locate the platform on more than one trial and were not tested further. In the subsequent probe trial, percentage time spent in the quadrant that previously held the platform was significantly higher than the opposite quadrant in *Trim67^+/+^* mice, however *Trim67^−/−^* mice failed to show quadrant selectivity and had lower percentage time than *Trim67^+/+^* in the target quadrant (genotype × quadrant interaction, *p* = 0.0343; Fig. 8*B*). *Trim67^−/−^* mice also demonstrated impairment in the number of crossings over the platform’s previous location [main effect of genotype, *p* = 0.0101, genotype × quadrant interaction, *p* = 0.0281].

During this reversal phase of testing to assess cognitive flexibility, *Trim67^−/−^* mice showed significant deficits in learning the new location of the escape platform (main effect of genotype, *p* = 0.0388; Fig. 8*C*). During the subsequent probe trial, only the *Trim67^+/+^* mice showed a preference for spending more time in the quadrant previously containing the platform in the reversal trials, while neither genotype displayed a significant difference in target crossings (Fig. 8*D*). Normal behaviors during the visible platform test and other sensory tests indicate TRIM67 is specifically required for appropriate spatial learning and memory and cognitive flexibility.

### Deficits in social novelty preference but not sociability

**Figure 9.**
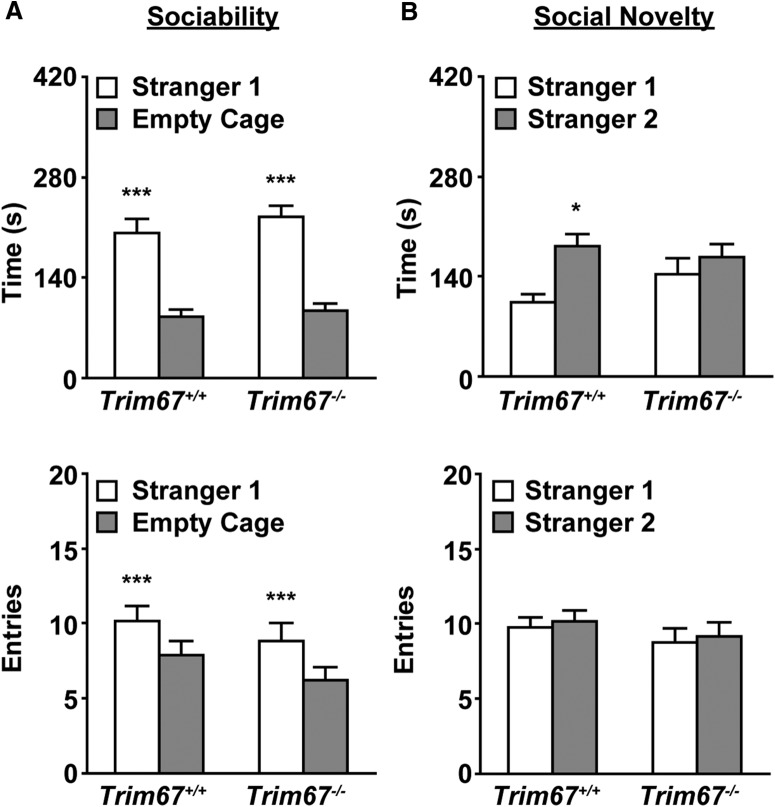
*Trim67^−/−^* mice display normal sociability, but impaired social novelty preference. ***A***, Measures of total time spent in (top) and entries into (bottom) side chambers of a three-chamber socialization assay, showing mice of both genotypes exhibited a significant preference for proximity to stranger mouse 1 (***p < 0.0001). ***B***, Total time spent in and entries into side chambers in subsequent three chamber social novelty assay. *Trim67^−/−^* mice show a lack of preference for the novel stranger, whereas *Trim67^+/+^* mice show a preference (*p = 0.0295).

Mice were evaluated for the effects of *Trim67* deficiency on social preference using a 3-chamber choice test. This behavior has been associated with the hippocampus and parts of the amygdala (Felix-Ortiz and Tye, 2014; Paine et al., 2017), both of which were decreased in size in *Trim67^−/−^* brains. In the initial habituation phase, both *Trim67^+/+^* and *Trim67^−/−^* mice made similar numbers of entries into each side chamber (*Trim67^+/+^*; 10.3 ± 1 right, 9.3 ± 0.7 left: *Trim67^−/−^*; 8.5 ± 0.9 right, 8.3 ± 0.8 left). In the test for sociability (Fig. 9*A*), both genotypes showed a preference for spending time in proximity to the cage containing stranger mouse 1 versus an empty cage. However, in the subsequent social novelty phase (Fig. 9*B*), *Trim67^−/−^* mice showed no preference for a novel stranger mouse, whereas *Trim67^+/+^* mice did. In both sociability and novelty phases, both groups of mice showed a similar number of entries into each side chamber, indicating that the lack of social novelty preference in the *Trim67^−/−^* group was not due to alterations in activity during the test. These data suggest that TRIM67 is required for a preference for social novelty but not for overall sociability.

### Impairments in sensorimotor gating

**Figure 10.**
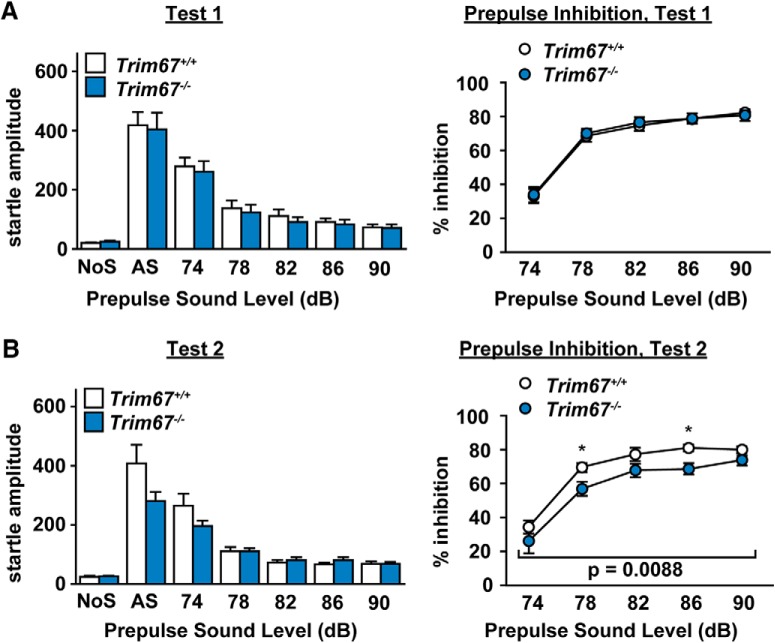
Prepulse inhibition of acoustic startle is impaired by loss of *Trim67*. ***A***, Startle amplitude (in arbitrary units) in response to no stimulus (NoS) or 120-dB stimulus (AS), with varying prepulse levels 100 ms before stimulus. ***B***, Prepulse inhibition is reported as % inhibition compared to AS. Test 1 took place at 10–11 weeks of age and test 2 at 17–19 weeks. *Trim67^−/−^* mice showed a decrease in prepulse inhibition in the second trial (*p* = 0.0088) but not in the first.

We hypothesized that the reduction in amygdala size in *Trim67^−/−^* mice may affect specific behaviors. Lesions of the amygdala have been associated with impairments in sensorimotor gating, specifically the basolateral region (Wan and Swerdlow, 1996; Howland et al., 2007). Mice were tested both at 10–11 weeks and at 17–19 weeks of age for prepulse inhibition of acoustic startle responses. At the first test, *Trim67^−/−^* mice showed no difference in either initial startle amplitude or in the % inhibition by prepulses of any intensity (Fig. 10*A*). However, in the retest at 17–19 weeks, *Trim67^−/−^* mice exhibited a mild decrease in startle response, and significant deficits in prepulse inhibition (Fig. 10*B*). This suggests impairments in sensorimotor gating emerged by the age of four months and that TRIM67 may function in the adult animal as well.

### *Trim67^−/−^* mice have impaired muscle function

**Figure 11.**
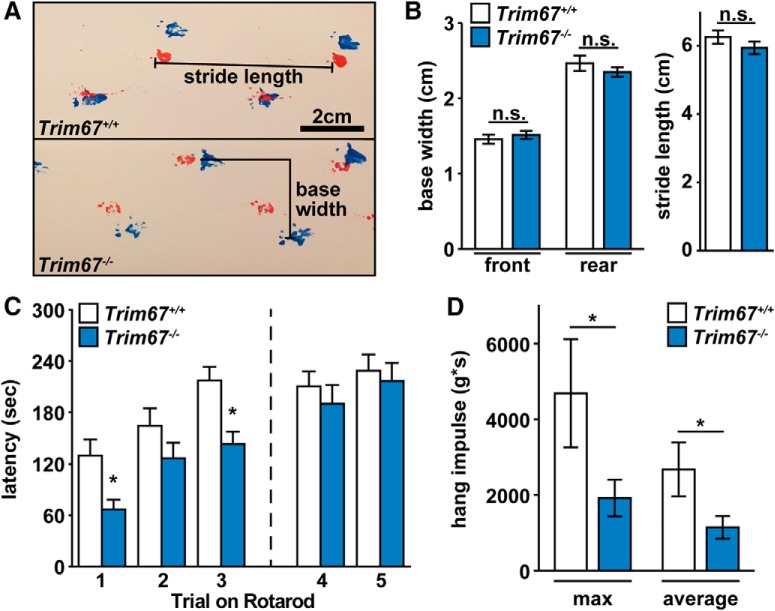
Muscle function, but not motor learning, is impaired in *Trim67^−/−^* mice. ***A***, Mouse gait was measured by footprint analysis on a straight track, with forepaws in red and hindpaws in blue. ***B***, There was no significant difference in base width (forepaws, *p* = 0.775; hindpaws, *p* = 0.229) or stride length (*p* = 0.252) during normal walking between *Trim67^−/−^* and *Trim67^+/+^*mice. ***C***, Latency to fall from an accelerating rotarod in three trials separated by 45 s (1, 2, 3), followed by two additional trials 48 h later separated by 45 s (4, 5). Deletion of *Trim67* led to a decrease in fall latency during the first day of trials (*p* = 0.0074); however, there was no difference during the retest trials. ***D***, Muscle tone measured during a four-paw rolling wire hang assay, reported as maximum and average impulse (weight × time) over three trials. *Trim67^−/−^* mice have lower maximum (*p* = 0.0387) and average (*p* = 0.0339) hanging impulse than *Trim67^+/+^*mice; *p < 0.05.

Mice carrying a spontaneous mutation in *Dcc* display overt impairments in motor function, including hopping behavior instead of a typical gait pattern (Finger et al., 2002; Welniarz et al., 2017). Thirteen *Trim67^+/+^*(five female, eight male) and thirteen *Trim67^−/−^* (eight female, five male) mice were assayed for gait and muscle function. Assessment of footprints on a linear track (Fig. 11*A*) indicated there were no differences between stride length or in either front or rear base width for *Trim67^+/+^* and *Trim67^−/−^* mice, suggesting no effect on gait (Fig. 11*B*). Subjects were tested for motor coordination and learning on an accelerating rotarod. In the initial day of testing (trials 1–3) *Trim67^−/−^* mice exhibited deficits in acquisition of motor learning (Fig. 11*C*), as indicated by a decreased latency to fall from the top of the rotating barrel. This difference was not observed on the second day of testing 48 h later (trials 4, 5). This suggests that deletion of *Trim67* leads to impairment of the initial performance, but not eventual learning, of a motor task. Since motor learning was not impaired, we suspected that decreased initial latency to fall may be due to impaired muscle function, and assayed muscle strength in a four-paw rolling-wire hang (Hoffman and Winder, 2016). The maximum and average hang impulse of three trials were significantly lower in *Trim67^−/−^* mice (Fig. 11*D*), suggesting an overall decrease in muscle tone.

### Altered striatal and thalamic anatomy in *Trim67^−/−^* mice

**Figure 12.**
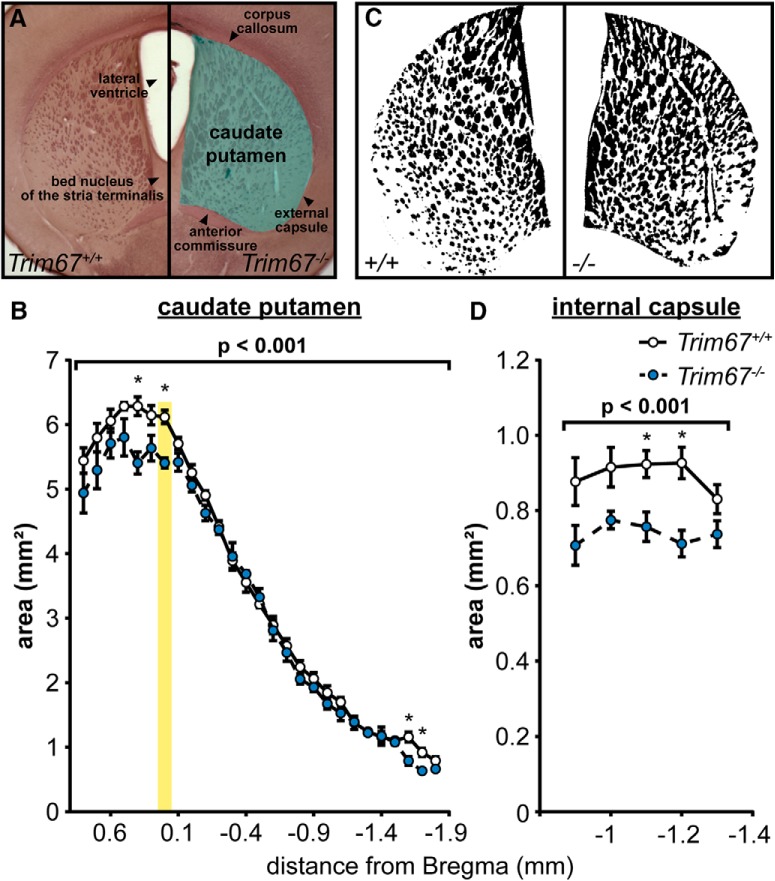
Malformation of CPu in *Trim67^−/−^* mice. ***A***, Photomicrographs of black-gold stained coronal brain sections showing the area used for quantification of CPu parameters. ***B***, The area of the CPu, reported as the average of both individual hemispheres. Yellow box denotes position of white matter measurements. The CPu was smaller in *Trim67^−/−^* brains (*p* = 0.00000876), with most significantly different individual positions toward the anterior. ***C***, Binary masks of white matter (black regions) in the CPu after semi-automated segmentation. ***D***, Deletion of *Trim67* resulted in a decrease in the area of the internal capsule, reported as the average of both hemispheres (*p* = 0.000138; *p <0.05 at this position).

In addition to circuits in the hippocampus and cortex, striatal circuits are implicated in spatial learning and memory and motor activity, particularly in the CPu (Oliveira et al., 1997; Dang et al., 2006; Lee et al., 2014). Since loss of *Trim67* disrupted these behaviors, we performed black gold staining of serial coronal sections through the CPu of five-week-old male and female littermates (Fig. 12*A*) to determine whether there were malformations of this region. This revealed that the CPu was smaller in *Trim67^−/−^* brains (Fig. 12*B*), which appeared to be largely in more anterior portions. We generated a binary mask of the myelin-stained CPu at bregma to determine whether the change in CPu was due to a loss of white and/or gray matter (Fig. 12*C*). Although there was no difference in total area of white matter between genotypes (*Trim67^+/+^* = 2.28 ± 0.12 mm^2^, *Trim67^−/−^* = 2.47 ± 0.12 mm^2^, *p* = 0.322, Mann–Whitney), when normalized to CPu area, white matter was increased in *Trim67^−/−^* brains (*Trim67^+/+^* = 38.15% ± 1.81%, *Trim67^−/−^* = 46% ± 2.23%, *p* = 0.030, Mann–Whitney), indicating a reduction in gray matter in *Trim67^−/−^* brains. Despite equal white matter in the striatum, the cross-sectional area of the internal capsule, where these fibers fasciculate, was smaller in *Trim67^−/−^* brains (Fig. 12*D*).

**Figure 13.**
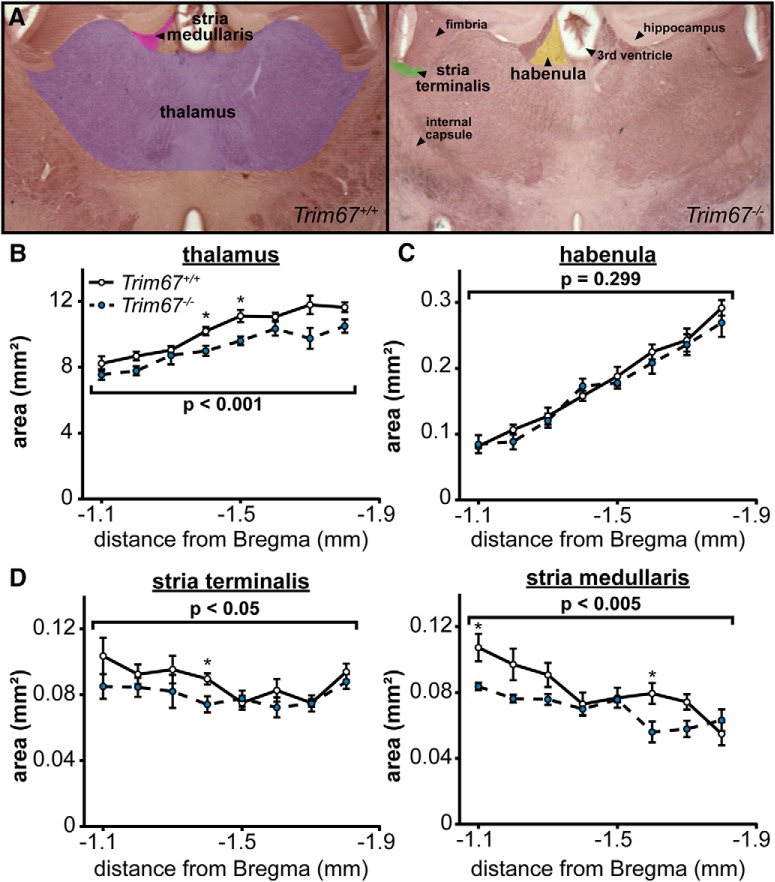
Deletion of *Trim67* is associated with a reduction in thalamus size. ***A***, Coronal brain sections with the thalamus, habenula, stria terminalis, and stria medullaris regions outlined as used for measurements. ***B***, *Trim67* deletion resulted in a decrease in the area of the thalamus in the region under the anterior hippocampus (*p* = 0.000174). ***C***, The area of the habenula was not affected by deletion of *Trim67* (*p* = 0.299). ***D***, Both the stria terminalis and stria medullaris were reduced in cross-sectional area in *Trim67^−/−^* brains (*p* = 0.0222 and *p* = 0.00152, respectively; *p < 0.05 at this position).

Deficits in sensorimotor gating have been associated with abnormalities in the sensory nuclei of the thalamus (Young et al., 1995; Campbell et al., 2007; Hazlett et al., 2008). In light of the sensorimotor gating disruption in *Trim67^−/−^* mice (Fig. 10) and TRIM67 expression the embryonic thalamus (Fig. 3*C*), we measured the size of the thalamus in five-week-old male and female littermates (Fig. 13*A*). Thalamic size was decreased in *Trim67^−/−^* mice (Fig. 13*B*). However, the neighboring nucleus of the habenula was the same size compared to *Trim67^+/+^* brains (Fig. 13*C*), indicating there was not hypotrophy of all diencephalic structures. Since the TRIM67 interaction partner DCC (Fig. 4*D*,*E*) is present in the thalamic and habenular associated fibers tracts of the stria medullaris and stria terminalis, respectively (Shu et al., 2000), we compared their cross-sectional area in serial coronal sections, to investigate whether these fiber tracts were malformed as was the cortical commissure of the corpus callosum. This revealed that the stria medullaris and stria terminalis were both smaller in cross-sectional area in *Trim67^−/−^* brains (Fig. 13*D*). These anatomic measurements demonstrate that deletion of *Trim67* results in decreases in the size of some, but not all, brain regions and fiber tracts in the adult.

## Discussion

Here, we generated a novel knockout mouse and specific antibody to investigate the largely unstudied, yet evolutionarily conserved E3 ubiquitin ligase, TRIM67. We show that TRIM67 is enriched in the cerebellum and, to a lesser extent, other brain regions in the adult mouse. Furthermore, in the embryo, TRIM67 is enriched in the developing cortex, diencephalon, and midbrain. We found that *Trim67* deletion results in a complex set of behavioral deficits and malformation of several brain regions and axonal fiber tracts in adult mice. In humans, the *TRIM67* gene, which thus far has not been linked to any human disorders, is within the q42.2 band of chromosome 1, a region highly associated with heritable neurologic conditions (St. Clair et al., 1990; Millar et al., 2000; Williams et al., 2009). The proximity of *TRIM67* to other genes linked to Alzheimer’s disease and schizophrenia (*DISC1*; Beecham et al., 2009; Carless et al., 2011), Parkinson’s disease (*SIPA1L2*; Nalls et al., 2014), and cognitive function (*DISC2*, *C1orf131*, *GALNT2*; Xu et al., 2017) by genome-wide association studies (GWAS), and the behavioral deficits revealed here associated with deletion of murine *Trim67* are intriguing, potentially suggesting possible involvement of *TRIM67* in human disorders or behaviors. Indeed, a small GWAS of patients with neuroticism, a personality trait that often occurs with major depression and anxiety disorders, identified variations in several genes, including *TRIM67* (Shifman et al., 2008). However, none of these variations reached genome wide significance levels, potentially due to the limited size of the study.

### A role for TRIM67 in brain development and maintenance

The change in TRIM67 protein levels from embryonic to adult brains suggests that TRIM67 could function both in developing neurons, for example the embryonic cortex, as well as mature neurons, such as cells of the adult cerebellum. Indeed, despite *Trim67* being deleted globally, sensorimotor gating deficits in prepulse inhibition of acoustic startle were not present at 10–11 weeks but appeared by 17–19 weeks. This suggests that TRIM67 may also be necessary for maintenance of brain function in mature animals. The spatial and motor learning impairments also suggest a role for TRIM67 in remodeling of the hippocampus, classically associated with the Morris water maze task (Clark et al., 2007). This study may support an intriguing possibility that the lack of TRIM67 may result in the onset of neurologic dysfunction with age, a common feature of many human disorders including schizophrenia, bipolar disorder, and neurodegenerative diseases (Larson and Nyman, 1970; Häfner et al., 1994; Carlson et al., 2002).

There is apparent hypotrophy of the hippocampus, striatum, thalamus, and amygdala in *Trim67^−/−^* brains which, based on black-gold staining, is due to decreased non-myelinated tissue. Given the normal size of the cortex and some portions of the affected nuclei, this may be due to abnormal maturation and development of specific types of neurons, as opposed to overall proliferation. This distinction will require additional studies to determine which neuron types express TRIM67, and at what points during development or maturity TRIM67 is expressed. The pattern of TRIM67 expression in the embryonic brain however supports a role for TRIM67 in neuronal development after proliferation, as TRIM67 protein levels are highest in regions of the developing cortex and hippocampus containing migrating and maturing, postmitotic neurons. This may offer an explanation for the reduction in adult gray matter, as a large portion of this tissue consists of neurites as opposed to cell bodies. A previous study on TRIM67 suggested a role in the formation and elongation of neurites (Yaguchi et al., 2012), and the similar protein TRIM9 regulates dendritic arborization in the hippocampus (Winkle et al., 2016), both of which support this possibility. The decreased gray matter observed in *Trim67^−/−^* mice alternatively may originate from decreases in branching of axons in the affected brain regions, whereas the cell bodies of the affected neurons reside in distant nuclei. As TRIM67 interacts with the axon guidance receptor DCC, which can promote axon branching (Dent et al., 2004; Winkle et al., 2014), this is an intriguing hypothesis, particularly since several DCC-expressing, netrin-sensitive axon tracts are malformed in the brains of *Trim67^−/−^* mice, including the callosal and hippocampal commissures, and the stria terminalis and stria medullaris.

These experiments have laid a foundation for investigating the role of TRIM67 in development and function of the brain. The role of TRIM67 ubiquitin ligase activity, its substrates, and the consequences of their ubiquitination remain to be identified. Further studies will be required to elucidate not only this molecular function of TRIM67 in developing and adult neurons, but also whether TRIM67 plays a role in DCC-dependent responses, the underlying cause of brain hypotrophy resulting from *Trim67* deletion, the changes in cellular structure that lead to fiber tract malformation, the time course of and underlying cause of behavioral abnormalities in *Trim67^−/−^* mice, and the contribution of *TRIM67* variations to human neurologic disorders. Furthermore, although TRIM67 is the most evolutionarily conserved vertebrate Class I TRIM, unlike the invertebrate TRIM ortholog, loss of *Trim67* does not fully phenocopy the axon tract defects seen in mice lacking *Dcc*. Future studies will need to examine whether this is due to compensation from other Class I TRIM proteins such as TRIM9, which interacts with both DCC and TRIM67, or TRIM1 and TRIM18, whose mutation in humans is associated with midline birth defects.

## Synthesis

Reviewing Editor: Karen Szumlinski, University of California at Santa Barbara Decisions are customarily a result of the Reviewing Editor and the peer reviewers coming together and discussing their recommendations until a consensus is reached. When revisions are invited, a fact-based synthesis statement explaining their decision and outlining what is needed to prepare a revision will be listed below. Note: If this manuscript was transferred from JNeurosci and a decision was made to accept the manuscript without peer review, a brief statement to this effect will instead be what is listed below.

I have reviewed the authors' rebuttal to their review by the Journal of Neuroscience and agree with the original Reviewer 1 that this report provides some very exciting new information regarding Trim67. The authors made a very conscientious effort to address the critiques of both reviewers and I agree with the authors that while additional experiments would obviously increase the impact of the work, such experiments would massively delay publication of important and new findings. In my opinion, the authors have addressed short-comings and future work sufficiently to warrant publication. As such, I have taken the liberty of accepting this transferred report for publication in eNeuro. I look forward to seeing this project advance in future work.
